# Supplementary material for: Predicting visual function by interpreting a neuronal wiring diagram
Source: Nature. 2024 Oct 2;634(8032):113–23. doi: 10.1038/s41586-024-07953-5 (PMC11446822; doi:10.1038/s41586-024-07953-5)
Supplement: Supplementary file 6 — CRF and ERF predictions for individual TmY4 and TmY9 cells. Analogous to Supplementary Data 3, but for TmY target types. Shown are the top four monosynaptic pathways, the strongest pathway passing through each of the top ten intermediary types (ranking from Extended Data Fig. 7), and the trisynaptic pathway Tm1–TmY–Dm3–TmY (see the section entitled Prediction of spatial normalization). [file 41586_2024_7953_MOESM6_ESM.zip › DataS4/TmY9qΓèÑ/720575940632788511.pdf]

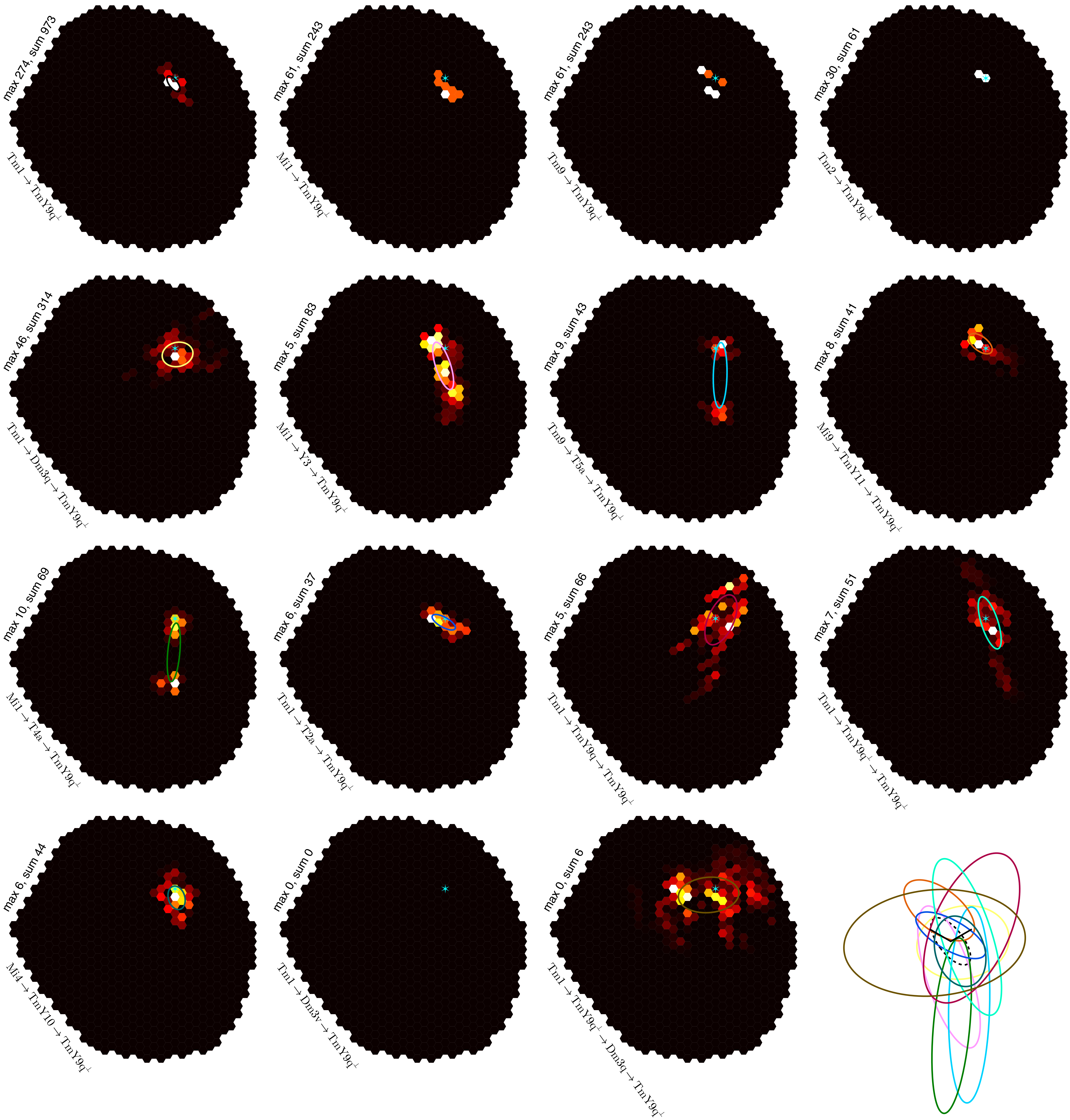
max 274, sum 973
Tm1 → TmY9q⊥
max 61, sum 243
Mi1 → TmY9q⊥
max 61, sum 243
Tm9 → TmY9q⊥
max 30, sum 61
Tm2 → TmY9q⊥
max 46, sum 314
Tm1 → Dm3q → TmY9q⊥
max 5, sum 83
Mi1 → Y3 → TmY9q⊥
max 9, sum 43
Tm9 → T5a → TmY9q⊥
max 8, sum 41
Mi9 → TmY11 → TmY9q⊥
max 10, sum 69
Mi1 → T4a → TmY9q⊥
max 6, sum 37
Tm1 → T2a → TmY9q⊥
max 5, sum 66
Tm1 → TmY9q → TmY9q⊥
max 7, sum 51
Tm1 → TmY9q⊥ → TmY9q⊥
max 6, sum 44
Mi4 → TmY10 → TmY9q⊥
max 0, sum 0
Tm1 → Dm3v → TmY9q⊥
max 0, sum 6
Tm1 → TmY9q⊥ → Dm3q → TmY9q⊥
